# Exploring genetic determinants of antimicrobial resistance in *Brucella melitensis* strains of human and animal origin from India

**DOI:** 10.3389/fmicb.2024.1474957

**Published:** 2024-10-04

**Authors:** Haris Ayoub, M. Suman Kumar, Rishabh Mehta, Prasad Thomas, Muskan Dubey, Himani Dhanze, Ganavalli S. Ajantha, K. N. Bhilegaonkar, Harith M. Salih, Charley A. Cull, Ravindra P. Veeranna, Raghavendra G. Amachawadi

**Affiliations:** ^1^Division of Veterinary Public Health, ICAR-Indian Veterinary Research Institute, Izatnagar, India; ^2^Division of Bacteriology and Mycology, ICAR-Indian Veterinary Research Institute, Izatnagar, India; ^**3**^Xavier University School of Medicine and Xavier University School of Veterinary Medicine, Oranjestad, Aruba; ^4^Department of Microbiology, SDM College of Medical Sciences and Hospital, Shri Dharmasthala Manjunatheshwara University, Dharwad, India; ^5^Department of Clinical Sciences, College of Veterinary Medicine, Kansas State University, Manhattan, KS, United States; ^6^Midwest Veterinary Services, Inc., Oakland, NE, United States

**Keywords:** brucellosis, antimicrobial susceptibility, efflux genes, single nucleotide polymorphism, *Brucella melitensis*

## Abstract

**Introduction:**

Antimicrobial resistance (AMR) in *Brucella melitensis*, the causative agent of brucellosis, is of growing concern, particularly in low and middle-income countries. This study aimed to explore the genetic basis of AMR in *B. melitensis* strains from India.

**Methods:**

Twenty-four isolates from humans and animals were subjected to antimicrobial susceptibility testing and whole-genome sequencing.

**Results:**

Resistance to doxycycline (20.80%), ciprofloxacin (16.67%), cotrimoxazole (4.17%), and rifampicin (16.67%) was observed. Genome analysis revealed efflux-related genes like *mprF*, *bepG*, *bepF*, *bepC*, *bepE*, and *bepD* across all isolates, however, classical AMR genes were not detected. Mutations in key AMR-associated genes such as *rpoB*, *gyrA*, and *folP* were identified, intriguingly present in both resistant and susceptible isolates, suggesting a complex genotype–phenotype relationship in AMR among *Brucella* spp. Additionally, mutations in efflux genes were noted in resistant and some susceptible isolates, indicating their potential role in resistance mechanisms. However, mutations in AMR-associated genes did not consistently align with phenotypic resistance, suggesting a multifactorial basis for resistance.

**Discussion:**

The study underscores the complexity of AMR in *B. melitensis* and advocates for a holistic multi-omics approach to fully understand resistance mechanisms. These findings offer valuable insights into genetic markers associated with AMR, guiding future research and treatment strategies.

## 1 Introduction

The genus *Brucella*, belonging to the bacterial family *Brucellaceae*, encompasses the causative agents of brucellosis, a globally significant zoonotic disease with an annual incidence exceeding 2.1 million cases in humans (Laine et al., 2023). Since the discovery of *Brucella melitensis* in 1887 by Sir David Bruce (Godfroid et al., 2000), brucellosis has remained a persistent threat.

Initially comprising six species with distinct mammalian host preferences—namely, *B. abortus, B. melitensis, B. suis, B. neotomae, B. ovis*, and *B. canis* (Corbel, 2006) – the genus has expanded to include 12 recognized species (Saavedra et al., 2019). Among these, *B. melitensis, B. suis*, and *B. abortus* pose significant zoonotic risks, with *B. canis* demonstrating lower zoonotic potential. Transmission to humans occurs through direct contact with infected animals, consumption of contaminated animal products, and inhalation of airborne agents, with unpasteurized milk and cheese serving as common sources of infection from sheep and goats (Pappas et al., 2005).

Members of *Brucella* species are Gram-negative coccobacilli or short rods with distinct morphological characteristics and a slow growth rate, necessitating specific culture conditions for isolation and identification (Alton et al., 1975; Pappas et al., 2005; Saavedra et al., 2019). Phenotypic identification methods, though traditional, may not always be conclusive due to high similarity among the strains of *Brucella* spp. However, molecular techniques such as Polymerase Chain Reaction (PCR)-based methods, including abortus, melitensis, ovis, and suis (AMOS)-PCR and Bruce ladder, have enhanced diagnostic accuracy (Bricker and Halling, 1994; López-Goñi et al., 2008, 2011). Furthermore, recent genomic and molecular studies have enhanced our understanding of *Brucella* species, distinguishing them from closely related genera such as *Ochrobactrum* at the genetic level. These studies emphasize the need for accurate species identification to avoid diagnostic errors and improve treatment outcomes (Moreno et al., 2023).

The genomic landscape of *Brucella* spp. members exhibits remarkable conservation across species, with a high degree of genetic similarity (>90%) (Michaux et al., 1993). The genome typically consists of two circular chromosomes encoding essential cellular functions distributed between them (Rajendhran, 2021). As facultative intracellular pathogens, *Brucella* species demonstrate the ability to survive and replicate within host cells, primarily macrophages, evading host immune responses and contributing to unique pathological features (Xavier et al., 2010; de Figueiredo et al., 2015).

Human brucellosis is a multisystemic disease, often involving various organs and presenting a diverse clinical spectrum, as highlighted in a landmark study from Kuwait, where 400 cases demonstrated the wide range of complications associated with the disease (Lulu et al., 1988). WHO guidelines recommend the use of antimicrobials doxycycline, rifampicin, streptomycin, cotrimoxazole, gentamicin, and ciprofloxacin individually or in combination for treatment of human brucellosis (Corbel, 2006), however, antimicrobial therapy is not recommended in animals. For uncomplicated brucellosis in adults, the first-line treatment is a combination of doxycycline and an aminoglycoside. Alternative regimens include doxycycline with rifampin or cotrimoxazole, while other oral options like quinolones may also be considered. In pediatric cases, studies have demonstrated the efficacy of using shorter treatment durations, including gentamicin for 5 days as part of combined therapy with other antimicrobials (Lubani et al., 1989). However, the emergence of AMR poses significant challenges to treatment efficacy (Trott et al., 2018). AMR studies in *Brucella* isolates from various regions have revealed instances of resistance to commonly used antimicrobials (Ilhan et al., 2013; Farazi et al., 2018; Liu et al., 2018; Elbehiry et al., 2022; Dadar et al., 2023). Relapse and treatment failures are also reported, highlighting the importance of periodic assessment of antimicrobial susceptibility profiles (Yousefi-Nooraie et al., 2012).

Understanding the genetic basis of AMR in *Brucella* is crucial for effective management and control of the disease. Whole genome sequencing (WGS) may offer insights into genomic factors underlying AMR, facilitating the identification of novel resistance markers and informing treatment strategies (Zankari et al., 2012; Khan et al., 2019). However, the molecular mechanisms of AMR in *Brucella* remain incompletely understood (Suárez-Esquivel et al., 2020; Wareth et al., 2021), warranting further investigation into the genomic aspects.

## 2 Materials and methods

### 2.1 Isolate retrieval and preparation

*B. melitensis* isolates used in this study were obtained from a collection of previously isolated strains received for confirmation and biotyping at the *Brucella* laboratory, Division of Veterinary Public Health, ICAR-IVRI. A total of 24 isolates from humans (*n* = 20), goat (*n* = 1) and sheep (*n* = 3) isolated from different regions of India over the period 2006–2023 were included. Primary cultivation of all isolates was carried out by inoculating the samples onto *Brucella* agar, and incubating under 10% CO_2_ at 37°C for upto 7 days. Isolates were characterized using Gram’s staining, biochemical tests and dye inhibition tests (Alton et al., 1988). Confirmation of species was done by AMOS PCR (Bricker and Halling, 1994). *B. melitensis* 16 M (ATCC 23456; NCTC 10094) was used as the reference strain.

### 2.2 Extraction of genomic DNA from isolates and PCR confirmation

Genomic DNA was extracted from the isolates using the snap-chill method for species-specific PCR confirmation. In the first step of bacterial lysate preparation, the culture was suspended in 200 μL of normal saline solution (NSS) and subjected to a 10-min heating in a boiling water bath, followed by rapid cooling on crushed ice. After this step, the prepared sample underwent centrifugation (utilizing the 2,326 K Hermle Labortechnik refrigerated centrifuge equipped with a 2 mL rotor) at 15,000 rpm at 4°C for 10 min. The resulting supernatant was then carefully collected in a fresh sterile tube and used as the DNA template for the subsequent PCR reaction mixture (Dashti et al., 2009).

For species confirmation, AMOS PCR was performed (Bricker and Halling, 1994). This multiplex PCR method uses five different primers, with one common reverse primer (IS711) and four forward primers, each specific to one of the following *Brucella* species: *B. abortus*, *B. melitensis*, *B. ovis*, and *B. suis*.

### 2.3 Antimicrobial susceptibility assay

All confirmed *B. melitensis* strains were scrutinized to determine their susceptibility to the selected antimicrobials. Isolates were tested for susceptibility against six different antimicrobials selected based on WHO guidelines for the treatment of human brucellosis *viz.*, doxycycline, rifampicin, streptomycin, cotrimoxazole, gentamicin, and ciprofloxacin (Corbel, 2006). MIC test (HiMedia test strips) was performed as per CLSI, 2021 guidelines. The antimicrobial content of all the MIC test strips used ranged between 0.001 and 240 (μg/mL), except for Doxycycline which ranged between 0.016 and 256 (μg/mL). The reference strain *E. coli* (ATCC 25922) was used as control. The Minimum Inhibitory Concentration (MIC) of the selected antimicrobials were determined on cation-adjusted Muller Hinton agar (CAMHA) plates with MIC test strips. Although CLSI recommends the use of Broth microdilution for antimicrobial susceptibility testing (AST) of *B. melitensis*, use of MIC gradient method/E-test with CAMHA has been implemented in other several other studies (Ma et al., 2023; Liu et al., 2018; Deshmukh et al., 2015; Khan et al., 2019; Dadar et al., 2023). *Brucella* cultures were grown on trypticase soy agar (TSA, BD BBL) and incubated for 48 h with or without CO_2_ depending on the requirement of the isolate. Inoculum was suspended in sterile NSS to match with 0.5 McFarland standard. The CAMHA plates were lawned with culture suspension within 15 min of its preparation and left to dry for about 15 min. MIC test strips were placed onto the inoculated CAMHA plates and incubated at 37°C for 42–48 h. All isolates were tested in duplicate to ensure reliability of results. MIC was taken as the value where the growth intersected the test strip. Interpretation of MIC was done for doxycycline, gentamicin, streptomycin and cotrimoxazole according to CLSI guidelines for potential bacterial agents of bioterrorism (CLSI, M45). As MIC breakpoints of ciprofloxacin and rifampicin are yet not established, MIC values were interpreted according to the CLSI guidelines for the fastidious bacterium *Haemophilus influenza* (CLSI, M100).

### 2.4 Extraction of genomic DNA for WGS

For the purpose of genome sequencing, isolates were subcultured onto Trypticase Soy agar and incubated at 37°C for 42 h. The genomic DNA of the 23 *B. melitensis* isolates (all study isolates except VPH-17-72) was extracted using QIAamp DNA Mini Kit (QIAGEN), following the manufacturer’s provided protocol using NFW as solvent. The concentration and purity of the extracted DNA were evaluated by measuring its optical density (OD) at 260 nm and 280 nm using a UV spectrophotometer (Eppendorf). The A260/A280 ratio was calculated to verify the DNA’s purity. The extracted DNA was sent in dry ice for WGS.

### 2.5 Genome assembly and annotation

The DNA samples were sent to miBiome Therapeutics LLP for genome sequencing. The genomes were sequenced with Illumina Miseq platform and sequencing data in the form of 150 bp paired end reads were obtained. The quality of the reads was assessed using FastP v0.23.2, an all-in-one preprocessing tool for FastQ files (Chen et al., 2018). The trimmed reads from FastP were denovo assembled in Unicycler v0.5.0 (Wick et al., 2017) constructing a set of contigs. The quality of the contigs was checked for the metrics such as N50 (contig length at which half of the genome is covered), contig size and number of uncalled bases (Ns) by QUAST v5.2.0 (Gurevich et al., 2013). The completeness of the genome assembly was evaluated using the Benchmarking Universal Single-Copy Orthologs (BUSCO v5.4.6) assessment tool (Simão et al., 2015).

The prokaryotic genome annotation tool (Prokka v 1.14.6) was used for gene prediction, functional annotation, and feature identification of final assembled sequence (Seemann, 2014). Most of the analysis was performed on Galaxy Europe^1^ (Afgan et al., 2018). The genome sequence of *B. melitensis* bv. 1 str. 16 M was used as reference. Further, the WGS of the isolate VPH-17-72 (Goat isolate, Accession no. GCA_003989885.1) was retrieved from NCBI database and used in the study.

### 2.6 Detection of ARGs

The genomic sequences were scanned against different databases such as Resfinder (Bortolaia et al., 2020), NCBI AMRFinderPlus (Feldgarden et al., 2021), ARG-ANNOT (Gupta et al., 2014), Megares (Doster et al., 2020) and CARD (Jia et al., 2016) using ABRicate to predict the presence of AMR genes (). ARGs with ≥90% identity were included in this study. Searches were also done with lower nucleotide similarity (60%) to widen the range of genes predicted.

### 2.7 SNP analysis and phylogeny

Whole genome alignment of the 24 study genomes was conducted using parSNP v1.2 (Treangen et al., 2014). The resulting multiple sequence alignment was subsequently employed for the construction of a phylogenetic tree for the 24 isolates using *B. melitensis* strain BwIM_SYR_04 (GCF_002191455.1) as reference. Using the Newick tree generated by parSNP as the starting tree, a maximum likelihood tree was created with RAxML-NG v1.2.0 (Kozlov et al., 2019). Statistical support in the form of bootstrap values was applied to assess the reliability of the branches within the phylogenetic tree. Bootstrap values close to 1 indicate strong support for the clades, while values close to zero suggest weaker support. Number of bootstrap replications was set to 200. The tree was visualized using iTOL (Letunic and Bork, 2016). Annotation of tree with relevant metadata was also done in iTOL. Using FASTA sequence alignment, generated with parSNP, number of SNPs were counted and pairwise SNP distance matrix was created with SNP-Dists v0.8.2.^2^

The aligned genomes were analyzed for SNPs comparing isolates with reference to AMR phenotypes obtained by the MIC test. SNPs calling in each isolate was done by SNIPPY v4.5.0.^3^ SNPs were generated using *B. melitensis* 16 M as reference. The selection of genes for SNP analysis was based on their potential association with reduced susceptibility to particular antimicrobials, including ciprofloxacin, gentamicin, streptomycin, sulfa-trimethoprim, and rifampicin. This selection was made based on observed intermediate susceptibility or resistance phenotypes among the tested strains. Attempts were made to map the varying MICs of the isolates with SNPs and the same was compared in terms of resistant/intermediate/susceptible phenotype. To further explore associations between observed AMR phenotypes and SNPs across the genomes, Genome-wide association studies (GWAS) were conducted using TreeWAS (Collins and Didelot, 2018).

### 2.8 SNP analysis of other genes potentially associated with antimicrobial resistance

SNP variants were also looked for in other genes that may be involved in AMR. These included the Small Multidrug Resistance (SMR) family, Multidrug and Toxic compound Extrusion (MATE) family, the Major Facilitator Superfamily (MFS), the ATP-Binding Cassette (ABC) family and the Resistance-Nodulation-cell Division (RND) family genes (Rahman et al., 2017). The RND family genes such as *bepC, bepD, bepE, bepF, bepG, mprF, mef, marC, emrE* and other genes related to efflux were studied.

## 3 Results

### 3.1 Confirmation of isolates

The confirmation of isolates was initially achieved through Gram stain and colony characteristics. Colonies displayed a smooth, convex, raised, and translucent appearance with an entirely intact edge. Microscopic examination revealed the presence of Gram-negative coccobacilli. Growth of isolates in presence of dyes classified all isolates as *B. melitensis*. Subsequently, confirmation was further established using AMOS PCR, which yielded distinctive bands at approximately 730 base pairs, specific for *B. melitensis*.

### 3.2 Phenotypic antimicrobial susceptibility of *Brucella melitensis* isolates

The interpretation of MIC values was carried out in accordance with the CLSI guidelines for potential bacterial agents of bioterrorism (CLSI, M45) for doxycycline, gentamicin, streptomycin and cotrimoxazole. However, since the MIC breakpoints for ciprofloxacin and rifampicin have not been formally established, MIC interpretation was done using the CLSI guidelines for fastidious bacterium *H. influenza*.

Based on MIC values from gradient strip method, all of the isolates exhibited susceptibility to gentamicin and streptomycin with a range in MIC values of 0.01–1 μg/mL and 0.01–3 μg/mL, respectively. Resistance to doxycycline, ciprofloxacin, cotrimoxazole and rifampicin was observed in 20.80, 16.67, 4.17 and 16.67% of the isolates, respectively. Two of the 24 (8.33%) isolates showed intermediate resistance to cotrimoxazole, as shown in Table 1.

**Table 1:** Results from MIC strip test for determining antimicrobial susceptibility of *B. melitensis* isolates.

Antimicrobial agent	Range	MIC_50_	MIC_90_	Degree of susceptibility (*B. melitensis*)					
	(μg/mL)	(μg/mL)	(μg/mL)	Susceptible		Intermediate		Resistant/Non-susceptible^#^	
				No.	%	No.	%	No.	%
Doxycycline	0.125–1.5	0.75	1.5	20	79.2	–	–	5	20.8
Ciprofloxacin	0.01–1.5	0.75	1.5	20	83.3	–	–	4	16.7
Gentamicin	0.01–1	0.1	0.25	24	100	–	–	0	0
Streptomycin	0.01–3	1	3	24	100	–	–	0	0
Rifampicin^*^	0.5–5	1	4	20	83.3	0	0	4	16.7
Cotrimoxazole^*^	0.01–5	0.1	2	21	87.5	2	8.3	1	4.17

^*^SIR pattern followed for Rifampicin and cotrimoxazole. –, # For doxycycline, ciprofloxacin, gentamicin and streptomycin, non-susceptible category is applicable.

### 3.3 Genome assembly

DNA extracted for sequencing had an average concentration of 19.485 ng/mL and a280/a260 of 1.74. Sequencing of *B. melitensis* isolates with Illumina Miseq platform provided 150 bp paired end reads. Sequencing data were received in FastQ format. Quality check revealed that all of the reads were of high quality with mean phred scores above 30 for all isolates. The filtered reads were used for genome assembly with Unicycler for all isolates, producing high quality draft genomes. The genome sizes of the 23 isolates exhibited diversity, with 22 isolates falling within a range of 3.262 Mb to 3.267 Mb, while one isolate had a smaller genome size of 3.09 Mb. The number of contigs in these isolates varied, with the majority having between 24 and 28 contigs, while one isolate displayed 69 contigs, reflecting differences in genome assembly quality. The genome fraction coverage among these isolates exhibited minor variability, ranging from 99.105 to 99.561%, with one isolate showing a lower genome fraction of 93.94%. Furthermore, the genome coverage values ranged widely, from 297.8 to 674.6, as shown in Supplementary Table S1.

### 3.4 Annotation

The number of CDS ranges from 2,958 to 3,131, indicating some variation that could be due to differences in gene content of the isolates. Three rRNA genes are constantly present in all isolates, typical for most bacterial genomes. The number of tRNA genes is mostly constant at 49. This constancy might suggest that these genes are highly conserved. A brief annotation summary of different isolates is given in Supplementary Table S2.

### 3.5 *In silico* identification of AMR genes

The ABRicate searches for AMR genes using different AMR databases yielded similar profiles in all of the isolates. The multiple peptide resistance factor (*Brucella_suis_*mprF) protein and RND-family efflux genes, *bep*C, *bep*D, *bepE*, *bep*F and *bep*G were identified by Megares with 98.87–99.84% identity. CARD yielded only the *Brucella_suis_*mprF and *triC* (a Triclosan specific efflux protein) genes. These genes were found in all of our isolates, irrespective of their phenotypic AMR profile. Details of genes identified are given in Table 2.

**Table 2:** Summary of identified AMR genes in isolates by ABRicate searches in AMR databases.

Type	Gene	Identity	Database
RND efflux pumps (Drug_and_biocide resistance)	*bepG*	99.66	MEGARES
	*bepF*	98.87	
	*bepC*	99.71	
	*bepD*	99.83	
	*bepE*	99.84	
Cationic_antimicrobial_peptides/ Defensin-resistant efflux pump	*mprF*	99.73	
Triclosan specific RND family efflux pump	*triC*	67.98	CARD

### 3.6 Phylogeny

**Figure 1:**
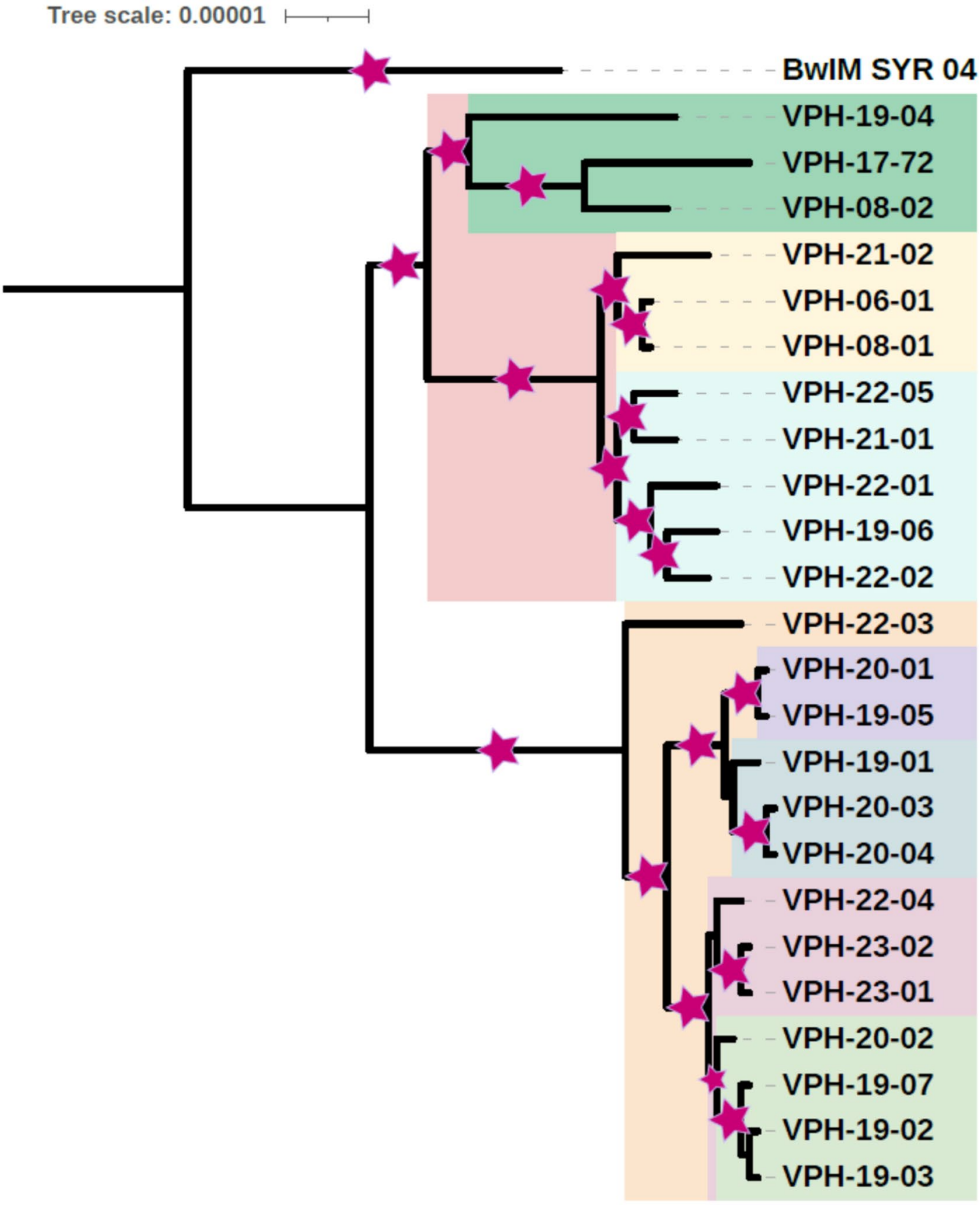
Core genome SNP based phylogenetic tree of *B. melitensis* isolates depicting place and year of isolation (middle two digits of isolate name) (Area of colored asterisk indicates bootstrap).

Phylogenetic tree was constructed using the 24 *B. melitensis* isolates from this study and the reference strain. Phylogenetic tree helped visualize the relationship between different *B. melitensis* isolates. Phylogeny based on SNPs revealed distinct groupings of isolates from India, suggesting geographical differentiation. The whole genome SNP based phylogeny was constructed using a Maximum Likelihood (ML) approach, and bootstrap support values were included to assess the robustness of the tree. For most of the branches bootstrap values were close to 1. The two bifurcations in the tree had bootstrap values, close to zero, 0 and 0.11 and one of the values was 0.59 as depicted in Figure 1.

**Figure 2:**
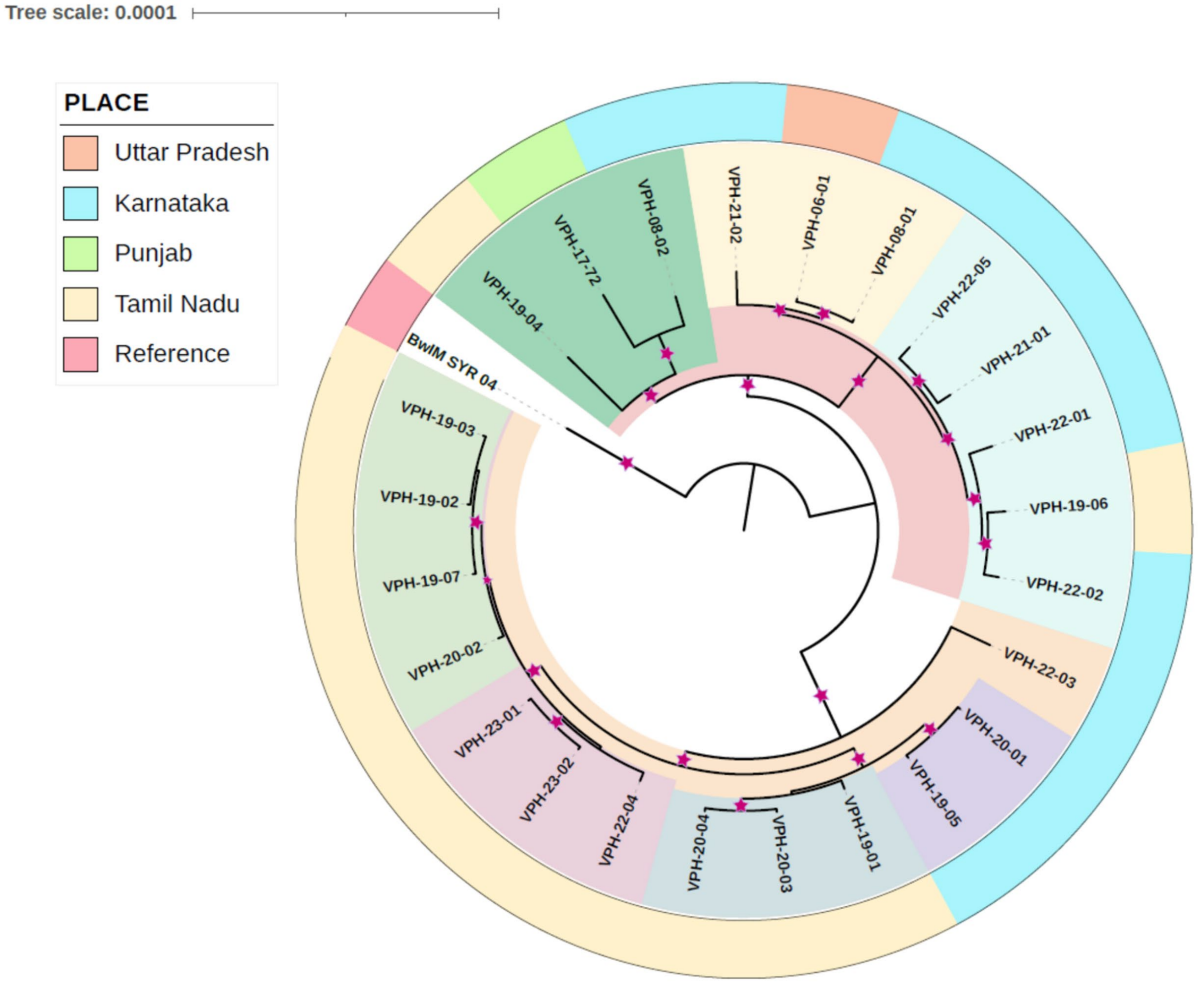
Core genome SNP based phylogetic tree indicating place and year of isolation. Relationship is indicated by color. Inner clades are indicated by shared color. The outer color strip denotes the place. The reference strain BwIM_SYR_04 is an outlier.

Based on the phylogenetic analysis, the study isolates were organized into two primary clusters. The isolates could be classified into five small phylogenetic groups, revealing limited genetic variation among them. Within the primary cluster, a distinct pattern emerged, with the majority of strains from Tamil Nadu exhibiting a close genetic relationship and clustering together. In contrast, isolates from Karnataka shared a common cluster with the Uttar Pradesh isolate. Interestingly, a separate clade was formed by specific isolates, including one from Tamil Nadu (VPH-19-04), one from Karnataka (VPH-08-02), and an isolate from Punjab, signifying a distinct genetic relationship. *B. melitensis* strain BwIM_SYR_04, serving as an outgroup, occupied a unique position outside of these clusters within the phylogenetic tree as illustrated in Figure 2.

### 3.7 SNP analysis in the genomes

The 24 sequenced genomes were analysed for SNP in all the genes relevant to AMR. Total number of SNPs in the study isolates compared to the reference genome were also analysed. Most of the isolates had similar SNPs in most of the coding DNA sequences. However, isolates also differed in SNPs in some of the genes. A total of 3,029 SNP sites were identified comparing our isolates to the reference genome *B. melitensis* 16 M, of which 740 were synonymous. Excluding the SNPs common to all of the study isolates, there were a maximum of 218 SNPs, some isolates even having a single SNP variation compared to the reference genome, indicating common origin. Some of the isolates were more closely related, having very few SNP differences as seen with the SNP distance matrix shown in Supplementary Figure S1.

### 3.8 SNP analysis in known and putative AMR genes and GWAS

The gene sequences for five known AMR associated genes *rpoB, folP*, g*yrA*, *gyrB* and *parC* were analysed for SNP variants comparing the sequence isolates to the *B. melitensis* 16 M reference genome. SNPs were observed only in four of the five genes compared to the reference genome. Only non-synonymous SNPs were considered for analysis relating to the role of genes in AMR. Four non-synonymous SNP variants were observed in *rpoB* gene, two in *folP*, two in *gyrA* and one in *parC* as shown in Table 3. No SNPs were detected in *gyrB* gene in any of the isolates. Mutations in the *rpo*B gene were common to all isolates, as such not limited to rifampicin resistant isolates. The *rpo*B SNPs cannot be associated with the resistant isolates. Mutations in *gyrA* gene were identified in two of four ciprofloxacin non-susceptibleisolates and also some susceptible isolates, as such not demonstrating a one-to-one correlation with AMR. SNPs in *folP* was detected in all cotrimoxazole non-susceptible isolates, and like with other genes, was not limited to this category. A number of SNPs were identified in the efflux genes *bepE*, *bepF*, *bepG* and *mprF,* common to all isolates, irrespective of their susceptibilityas shown in Table 3. However, only two of the observed SNP variations in efflux genes, *mprF* and *bepG* were non-synonymous and confined to only a subset of isolates, as depicted in Table 4. Mapping of AMR phenotype (resistant/intermediate/susceptible) of a particular isolate to an antimicrobial, to related AMR and efflux gene did not reveal a direct correlation between susceptibility and AMR/efflux gene presence and mutations, thereby, pointing to a complex genetic landscape of AMR for *B. melitensis.* GWAS analysis using TreeWAS corroborated these findings, showing no significant associations between the observed AMR phenotypes and the SNPs across the isolates.

**Table 3:** Summary of non-synonymous SNPs in AMR associated genes of study isolates.

Gene	Nucleotide position	Amino acid (position)
*rpoB*	1886\u00B0C > T	ALA>VAL (629)
	2,954\u00B0C > T	ALA>VAL (985)
	4,078 A > G	THR > ALA (1360)
	4,186 A > G	ASN > ASP (1396)
*gyrA*	1,195\u00B0C > T	PRO>SER (399)
	1735\u00B0C > G	LEU > VAL (579)
*parC*	1,040\u00B0C > T	Pro>Leu (347)
*folP*	631 T > C	PHE > LEU (211)
	448\u00B0C > G	ARG > GLY (150)
*bepE*	3043_3045delACTinsGCC	THR > ALA (1015)
	2,807 T > C	VAL > ALA (936)
	2,155 T > A	TYR > ASN (719)
	838 A > G	ASN > ASP (280)
*bepF*	920\u00B0C > A	THR > LYS (307)
	1,072 A > G	THR > ALA 358
*bepG*	1,031 T > C	ILE > THR (344)
	587\u00B0C > T	Ser > Phe (196)
*mprF*	253 G > C	Ala>Pro (85)

**Table 4:** SNP variations observed in AMR genes and phenotype (S = susceptible, I = intermediate, R = Resistant, NS = Nonsusceptible = NS; + = SNP present, − = SNP absent).

Lab id	DOX	CIP	COT	GEN	STM	RIF	*gyrA*	*folP*	*parC*	*mprF*	*bepG*
SNP position							1195C > T	448C > G	1040C > T	253G > C	587C > T
VPH-06-01	S	S	S	S	S	S	−	−	−	+	−
VPH-08-01	S	S	S	S	S	S	−	−	−	+	−
VPH-08-02	S	S	S	S	S	S	−	−	+	+	−
VPH-17-72	S	S	S	S	S	S	−	−	−	+	−
VPH-19-01	S	S	S	S	S	S	+	+	−	−	+
VPH-19-02	NS	NS	I	S	S	S	+	+	−	−	+
VPH-19-03	S	S	I	S	S	S	+	+	−	−	+
VPH-19-04	S	S	S	S	S	S	−	−	−	+	−
VPH-19-05	S	S	S	S	S	S	+	+	−	−	+
VPH-19-06	NS	S	S	S	S	S	−	−	−	+	−
VPH-19-07	S	S	R	S	S	R	+	+	−	−	+
VPH-20-01	S	S	S	S	S	R	+	+	−	−	+
VPH-20-02	S	S	S	S	S	R	+	+	−	−	+
VPH-20-03	S	S	S	S	S	S	+	+	−	−	+
VPH-20-04	S	NS	S	S	S	S	+	+	−	−	+
VPH-21-01	S	NS	S	S	S	S	−	−	−	+	−
VPH-21-02	NS	NS	S	S	S	S	−	−	−	+	−
VPH-22-01	NS	S	S	S	S	R	−	−	−	+	−
VPH-22-02	S	S	S	S	S	S	−	−	−	+	−
VPH-22-03	NS	S	S	S	S	S	+	+	−	−	+
VPH-22-04	S	S	S	S	S	S	+	+	−	−	+
VPH-22-05	S	S	S	S	S	S	−	−	−	+	−
VPH-23-01	S	S	S	S	S	S	+	+	−	−	+
VPH-23-02	S	S	S	S	S	S	+	+	−	−	+

## 4 Discussion

The current study investigated the *in vitro* susceptibility of 24 *B. melitensis* strains by gradient strip method against six antimicrobials commonly used for treatment of brucellosis, revealing that strains under study were mostly susceptible. However, non susceptible strains were not uncommon. Results indicated non susceptibility in 5/24 (20.83%) isolates to doxycycline with an MIC range of 0.125–1.5 μg/mL. Isolates with MIC >1 have been considered non-susceptible to doxycycline. Most of the previous studies have recorded 100% susceptiblity of *B. melitensis* to doxycycline (Deshmukh et al., 2015; Johansen et al., 2018; Khan et al., 2019; Gültekin et al., 2021; Wareth et al., 2021; Elbehiry et al., 2022; Dadar et al., 2023; Ma et al., 2023).

The isolates under study showed 100% susceptibility to streptomycin and gentamicin by gradient strip method. Similar susceptibility pattern was recorded in Norway (Johansen et al., 2018), Egypt (Wareth et al., 2021), China (Ma et al., 2023) and Iran (Dadar et al., 2023). Resistant isolates were also observed for ciprofloxacin (16.67%), cotrimoxazole (4.17%) and rifampicin (16.67%) in our study. Doimari et al. (2018) reported resistance to rifampicin (31.25%) and cotrimoxazole (37.5%) in *B. melitensis* isolates from India. In another study, Elbehiry et al. (2022) reported resistance to cotrimoxazole and rifampicin in 36.36 and 31.82% isolates, respectively. Khan et al. (2019) reported resistance to ciprofloxacin, rifampicin and streptomycin in 76.2, 66.7 and 4.8% *B. melitensis* isolates, respectively, indicating higher resistance.

Genome sequencing followed by assembly generated between 24 and 28 contigs with average genome size of 3,235,083 bp. This is near to the average genome size of *B. melitensis* previously reported (Salmon-Divon et al., 2018). Prokka annotation results for the *B. melitensis* isolates were consistent in terms of genome size and number of coding sequences and essential genes like rRNA and tRNA. The variability observed in the number of contigs and CDS may result from differences in the completeness of the assemblies or might be part of the natural genetic variation among the isolates. These results are close to well-annotated genomes; providing a robust foundation for further comparative genomic studies or functional analysis of *B. melitensis*. The whole genome SNP-based phylogenetic tree analysis of *B. melitensis* isolates revealed distinct groupings that may point to geographical differentiation within India. These findings are consistent with the observations made by Karthik et al. (2021) among 17 *B. melitensis* isolates from India. The clear separation of isolates into different clades highlights the role of geographic factors in shaping the genetic diversity of these isolates [46]. The analysis revealed two primary clusters within Indian isolates. The majority of strains from Tamil Nadu clustered closely together, highlighting a high degree of genetic relatedness. In contrast, isolates from Karnataka shared a cluster with a Uttar Pradesh isolate, that maybe due to a potential inter-state movement of strains. Additionally, a separate clade was formed by specific isolates from Tamil Nadu, Karnataka, and Punjab, showcasing the presence of distinct genetic lineages. Furthermore, the presence of isolates from the same region in different clades implied a history of evolutionary divergence and geographic expansion, shedding light on the complexity of *B. melitensis* evolution within India.

In-silico analysis was conducted for AMR in 24 *Brucella* isolates to gain insights into the genetic factors influencing resistance. Genomic investigation involved SNP analysis in genes reported to be associated with AMR in *Brucella* spp. to better understand the role of individual SNPs in resistance to particular antimicrobials. Additionally, whole genome SNP analysis was performed to understand the genomic structure of Indian *B. melitensis* isolates compared to the *B. melitensis* 16 M reference strain.

Several AMR genes, including *rpo*B, *fol*P, *gyr*A, *gyr*B, and *par*C, were identified in the isolates. However, it is essential to emphasize that the mere presence of these genes does not confirm antimicrobial resistance, a finding that is consistent with previous studies. For instance, the presence of *fol*P, a gene known to potentially explain resistance to cotrimoxazole (Pereira et al., 2023), was observed in all isolates from this study. The comprehensive search for AMR genes across multiple databases consistently revealed the presence of specific resistance determinants in all isolates. Notably, key efflux pump components, including the multiple peptide resistance factor (*Brucella*_*suis*_*mpr*F) protein and RND-family efflux genes (*bep*C*, bep*D*, bep*E*, bep*F, and *bep*G) were identified. These genes displayed a remarkable degree of sequence identity with reference sequences, underscoring their conservation within the genus. However, despite their known involvement in conferring resistance to antimicrobials, their specific roles in AMR in *Brucella* spp. remain enigmatic. These findings emphasize the need for further research to elucidate the mechanisms by which these efflux pumps contribute to AMR, shedding light on potential targets for the development of more effective strategies against *Brucella* infections (Ma et al., 2023). Crucially, our findings indicated that the presence of these AMR genes was ubiquitous among all isolates, irrespective of their phenotypic AMR profiles. As such mere presence of these genes may not be associated with resistance (Biswas et al., 2008). A number of putative efflux drug transporters belonging to ABC, RND, MFS & MATE families were identified and SNPs analysis revealed no non-synonymous SNPs in these genes. The SNP analysis of the entire genomes provided a deeper understanding of the genetic relatedness among the isolates. Although the majority of the isolates exhibited a high degree of similarity in SNP profiles, some differences were observed in certain genes. A total of 3,029 SNP sites were identified when comparing the isolates to the reference genome *B. melitensis* 16 M. These SNPs might represent potential markers for distinguishing different strains and understanding their evolutionary relationships (Tan et al., 2015).

In the context of the study on *B. melitensis*, a comprehensive analysis of AMR genes and Single Nucleotide Polymorphisms (SNPs) was conducted to better understand the complex nature of AMR in this pathogen. The findings of this study align with previous researches, revealing a multifaceted landscape of resistance mechanisms (Biswas et al., 2008; Dadar et al., 2023). Mutations were identified in the *rpo*B gene, which is known to be associated with rifampicin resistance (Khan et al., 2019). Two of the four mutations, 629-Ala (GCG)◊Val (GTG) and 985-Ala (GCC)◊Val (GTC) were also observed by (Johansen et al., 2018). However, these mutations were not exclusive to rifampicin-resistant isolates, questioning the exclusive role of *rpo*B gene mutations in conferring resistance to rifampicin in *B. melitensis* isolates. Similarly, (Marianelli et al., 2006) identified several *rpoB* mutations that served as molecular markers for genotyping *Brucella* spp. and were associated with rifampin resistance. For example, *B. abortus* RB51 was found to carry a D526Y (GAC > TAC) mutation, associated with the rifampin-resistant phenotype. Similarly, their study identified species-and biovar-specific mutations in *rpoB*, such as E270K (GAG>AAG) and N344D (AAC > GAC) in *B. abortus*, as well as V271A (GTC > GCC) in *B. suis* (Marianelli et al., 2006).

In our study, we identified *rpoB* mutations at positions 629 (ALA>VAL, GCG > GTG) and 985 (ALA>VAL, GCC > GTC), which were present across all our isolates, irrespective of their rifampicin susceptibility profile. While these mutations overlap with those described by Johansen et al. (2018) and align with some of the mutations described by Marianelli et al. (2007), the exact role of these mutations in conferring rifampicin resistance remains unclear. As previously noted by Marianelli et al. (2007) some mutations, such as L670F (CTT > TTT) in *B. melitensis*, were not linked to the development of rifampin resistance. In our analysis, similar complexities were observed, as *rpoB* mutations did not always correlate with rifampicin resistance phenotypes in the isolates, echoing the findings of Marianelli et al. (2007). These results suggest that, while specific *rpoB* mutations may serve as useful genotypic markers for *Brucella* species and biovars, their contribution to rifampicin resistance is not always straightforward. The role of efflux pumps in conferring resistance to rifampicin needs to be further explored. Mutations in the chromosomal *fol*P gene, previously linked to sulfonamide resistance (Martin et al., 2009), were detected in all non-susceptible (R/I) isolates and in some susceptible isolates, highlighting the complex nature of sulfonamide resistance. Mutations in the *gyr*A gene were identified in two out of four ciprofloxacin-resistant isolates and were also present in some susceptible isolates. These *gyr*A mutations are associated with the regulation of ciprofloxacin susceptibility (Khan et al., 2019). The efflux genes, particularly *bep*DE in the RND family, have been known to mediate resistance to tetracycline and fluoroquinolones, including ciprofloxacin. This suggests that the observed mutations in efflux genes may have a role in ciprofloxacin resistance by facilitating the extrusion of ciprofloxacin from bacterial cells. However, the presence of *gyr*A mutations in some susceptible isolates introduces complexity into our understanding of ciprofloxacin resistance mechanisms, warranting further exploration of the genetic factors contributing to ciprofloxacin resistance. In the context of doxycycline resistance, none of the *tet* genes were identified. Instead, mutations in efflux genes were prevalent in all doxycycline resistant isolates, and also some susceptible isolates. Efflux genes, such as *bep*DE in the RND family of *B. suis*, have been associated with resistance to tetracycline and fluoroquinolones, and mutations in the *bep*C gene have been linked to resistance to fluoroquinolones and aminoglycosides (Griffith et al., 2018). As such, observed mutations may partly explain the doxycycline resistance but needs further elucidation. The analysis revealed several novel SNPs within the aforementioned genes. Interestingly, two mutations, 985-Ala (GCC)◊Val (GTC) in the *rpo*B gene and 211-Phe (TTC)◊Leu (CTC) in the *fol*P gene at position 631, were consistently present across all isolates. These mutations have also been reported by Johansen et al. [40] in all isolates within the East Mediterranean clade. This shared occurrence implies their potential significance in the evolutionary dynamics particular to this clade.

The findings of this study emphasize the intricate nature of AMR in *B, melitensis*. AMR did not always correlate with the presence of specific genes or SNPs. In relation to resistance to specific antimicrobials, none of the mutations identified in the analyzed genes demonstrated a discernible concordance with the observed variations in antimicrobial susceptibility, thereby, highlighting the multifactorial basis of resistance in this pathogen. Furthermore, publicly available AMR databases may not comprehensively identify the genes responsible for resistance (Wareth et al., 2021). Further, analyzing individual genes in isolation may not suffice to fully understand the intricate genetic interactions that underlie AMR in *B. melitensis*.

In conclusion, our study provides valuable insights into the genetic landscape of *Brucella* isolates, particularly concerning AMR. While the consistent presence of certain AMR genes, such as the multiple peptide resistance factor (*Brucella_suis_mprF*) protein and RND-family efflux genes (*bepC, bepD, bepE, bepF,* and *bepG*), was observed across all isolates, it is important to note that these genes alone do not confirm phenotypic resistance. Previous studies have shown that these genes are widely distributed among *Brucella* species and across different geographic regions without necessarily correlating with AMR. This underscores the limitation of relying solely on genomic data to understand resistance in *Brucella*.

Our research highlights the necessity of integrating phenotypic testing with genetic analysis to develop more effective treatment strategies. The mutations identified in genes previously implicated in AMR did not always correspond with the anticipated phenotype–genotype relationship among the analyzed isolates. Additionally, insights gained from whole-genome sequencing shed light on the genomic epidemiology of *B. melitensis* strains in India, revealing evident geographic clustering and evolutionary divergence. Further investigations are needed to explore the full landscape of AMR in *Brucella*, emphasizing a multifactorial approach to comprehensively understand and combat this persistent zoonotic threat.

## Data Availability

The Whole Genome Sequences of the study isolates have been deposited in GenBank with relevant metadata under accession nos. JAYWOX000000000 (VPH-06-01), JAY-WOY000000000 (VPH-08-01), JAYWOZ000000000 (VPH-08-02), JAYWPA000000000 (VPH-19-01), JAYWPB000000000 (VPH-19-02), JAYWPC000000000 (VPH-19-03), JAYWPD000000000 (VPH-19-04), JAYWPE000000000 (VPH-19-05), JAYWPF000000000 (VPH-19-06), JAYWPG000000000 (VPH-19-07), JAYWPH000000000 (VPH-20-01), JAYWPI000000000 (VPH-20-02), JAY-WPJ000000000 (VPH-20-03), JAYWPK000000000 (VPH-20-04), JAYWPL000000000 (VPH-21-01), JAYWPM000000000 (VPH-21-02), JAYWPN000000000 (VPH-22-01), JAYWPO000000000 (VPH-22-02), JAYWPP000000000 (VPH-22-03), JAYWPQ000000000 (VPH-22-04), JAYWPR000000000 (VPH-22-05), JAYWPS000000000 (VPH-23-01) and JAYWPT000000000 (VPH-23-02).

## Footnotes

^1^usegalaxy.org

^2^https://github.com/tseemann/snp-dists

^3^https://github.com/tseemann/snippy
