# Supplementary material for: Exploring genetic determinants of antimicrobial resistance in Brucella melitensis strains of human and animal origin from India
Source: Front Microbiol. 2024 Oct 4;15:1474957. doi: 10.3389/fmicb.2024.1474957 (PMC11488214; doi:10.3389/fmicb.2024.1474957)
Supplement: Supplementary file 1 [file Data_Sheet_1.docx]

**Supplementary Figure. 1.** SNP distance matrix of study isolates and *B. melitensis* 16M (Ref.) indicating number of SNP variations in the individual isolates.
