# Supplementary material for: Exploring genetic determinants of antimicrobial resistance in Brucella melitensis strains of human and animal origin from India
Source: Front Microbiol. 2024 Oct 4;15:1474957. doi: 10.3389/fmicb.2024.1474957 (PMC11488214; doi:10.3389/fmicb.2024.1474957)
Supplement: Supplementary file 2 [file Data_Sheet_2.docx]

**Supplementary Figure. 1.** SNP distance matrix of study isolates and *B. melitensis* 16M (Ref.) indicating number of SNP variations in the individual isolates.

**Table S1:** Genome summary of study isolates

| **Isolate ID** | **Total reads (M)** | **Filtered reads (M)** | **Genome Size (Mb)** | **No. contigs** | **N50** | **Genome fraction** | **Genome coverage** | **GC Content (%)** |
| --- | --- | --- | --- | --- | --- | --- | --- | --- |
|  |  |  |  |  |  |  |  |  |
| VPH-06-01 | 7.347044 | 7.15822 | 3266318 | 26 | 249280 | 99.561 | 332.5 | 57.22 |
| VPH-08-01 | 6.596996 | 6.410628 | 3266346 | 25 | 249280 | 99.561 | 297.8 | 57.22 |
| VPH-08-02 | 6.681382 | 6.527074 | 3262454 | 69 | 91417 | 99.394 | 303.4 | 57.22 |
| VPH-19-01 | 11.298194 | 11.199012 | 3267449 | 25 | 276315 | 99.169 | 557.3 | 57.25 |
| VPH-19-02 | 11.943824 | 11.836264 | 3267511 | 24 | 293115 | 99.168 | 307.1 | 57.25 |
| VPH-19-03 | 6.416248 | 6.238506 | 3081237 | 25 | 249285 | 93.937 | 674.6 | 57.22 |
| VPH-19-04 | 14.560218 | 14.313068 | 3265202 | 27 | 293077 | 99.105 | 583.2 | 57.25 |
| VPH-19-05 | 12.62483 | 12.38699 | 3267506 | 24 | 293091 | 99.171 | 585.3 | 57.25 |
| VPH-19-06 | 12.559194 | 12.426234 | 3266414 | 25 | 293148 | 99.143 | 563.1 | 57.25 |
| VPH-19-07 | 12.068372 | 11.959162 | 3267570 | 24 | 293115 | 99.168 | 495.3 | 57.25 |
| VPH-20-01 | 10.706906 | 10.519896 | 3267482 | 24 | 293091 | 99.171 | 670.3 | 57.25 |
| VPH-20-02 | 14.377688 | 14.236112 | 3267531 | 24 | 293099 | 99.168 | 485.1 | 57.25 |
| VPH-20-03 | 10.443702 | 10.298964 | 3266316 | 33 | 193986 | 99.135 | 636.2 | 57.25 |
| VPH-20-04 | 13.697438 | 13.511842 | 3267499 | 24 | 293100 | 99.171 | 436.0 | 57.25 |
| VPH-21-01 | 9.389988 | 9.257616 | 3266511 | 25 | 293140 | 99.146 | 596.0 | 57.25 |
| VPH-21-02 | 12.795494 | 12.650802 | 3266302 | 26 | 249289 | 99.143 | 648.2 | 57.25 |
| VPH-22-01 | 13.931106 | 13.763442 | 3266422 | 25 | 293168 | 99.146 | 492.6 | 57.25 |
| VPH-22-02 | 10.566106 | 10.458546 | 3266556 | 25 | 293147 | 99.146 | 469.3 | 57.25 |
| VPH-22-03 | 10.114012 | 9.966226 | 3267414 | 24 | 293076 | 99.166 | 463.7 | 57.25 |
| VPH-22-04 | 9.959466 | 9.845878 | 3267343 | 24 | 293092 | 99.165 | 597.8 | 57.25 |
| VPH-22-05 | 12.842948 | 12.69338 | 3266389 | 25 | 293164 | 99.144 | 515.1 | 57.25 |
| VPH-23-01 | 11.036014 | 10.940654 | 3267421 | 24 | 293091 | 99.168 | 647.2 | 57.25 |
| VPH-23-02 | 13.860822 | 13.745732 | 3267421 | 24 | 293091 | 99.168 | 332.5 | 57.25 |

**Table S2:** PROKKA annotation summary for isolates

| **Isolate ID** | **Contigs** | **Bases** | **Repeat region** | **CDS** | **rRNA** | **tRNA** |
| --- | --- | --- | --- | --- | --- | --- |
| VPH-06-01 | 26 | 3281401 | 0 | 3124 | 3 | 49 |
| VPH-08-01 | 25 | 3281413 | 0 | 3127 | 3 | 49 |
| VPH-08-02 | 69 | 327756 | 0 | 3131 | 3 | 47 |
| VPH-19-01 | 25 | 3286144 | 0 | 3126 | 3 | 49 |
| VPH-19-02 | 24 | 3285978 | 0 | 3127 | 3 | 49 |
| VPH-19-03 | 25 | 3095737 | 0 | 2958 | 3 | 48 |
| VPH-19-04 | 27 | 3285052 | 0 | 3125 | 3 | 49 |
| VPH-19-05 | 24 | 3286179 | 0 | 3127 | 3 | 49 |
| VPH-19-06 | 24 | 3284884 | 0 | 3125 | 3 | 49 |
| VPH-19-07 | 25 | 3285744 | 0 | 3124 | 3 | 49 |
| VPH-20-01 | 24 | 3286163 | 0 | 3127 | 3 | 49 |
| VPH-20-02 | 24 | 3285702 | 0 | 3125 | 3 | 49 |
| VPH-20-03 | 33 | 3284996 | 0 | 3130 | 3 | 49 |
| VPH-20-04 | 24 | 328618 | 0 | 3126 | 3 | 49 |
| VPH-21-01 | 25 | 3285103 | 0 | 3124 | 3 | 49 |
| VPH-21-02 | 26 | 3284773 | 0 | 3127 | 3 | 49 |
| VPH-22-01 | 25 | 3285166 | 0 | 3127 | 3 | 49 |
| VPH-22-02 | 25 | 3285303 | 0 | 3121 | 3 | 49 |
| VPH-22-03 | 24 | 3285712 | 0 | 3123 | 3 | 49 |
| VPH-22-04 | 24 | 3285515 | 0 | 3127 | 3 | 49 |
| VPH-22-05 | 25 | 3284877 | 0 | 3121 | 3 | 49 |
| VPH-23-01 | 24 | 3286028 | 0 | 3123 | 3 | 49 |
| VPH-23-02 | 24 | 3285688 | 0 | 3122 | 3 | 49 |
